# Impact of intercensal population projections and error of closure on breast cancer surveillance: examples from 10 California counties

**DOI:** 10.1186/bcr1266

**Published:** 2005-06-07

**Authors:** Amanda I Phipps, Christina A Clarke, Rochelle R Ereman

**Affiliations:** 1 Marin County Department of Health and Human Services, Epidemiology Program, San Rafael, California, USA; 2 Currently working at the Northern California Cancer Center, Fremont, California, USA; 3 Northern California Cancer Center, Fremont, California, USA

## Abstract

**Introduction:**

In 2001, data from the California Cancer Registry suggested that breast cancer incidence rates among non-Hispanic white (nHW) women in Marin County, California, had increased almost 60% between 1991 and 1999. This analysis examines the extent to which these and other breast cancer incidence trends could have been impacted by bias in intercensal population projections.

**Method:**

We obtained population projections for the year 2000 projected from the 1990 census from the California Department of Finance (DOF) and population counts from the 2000 US Census for nHW women living in 10 California counties and quantified age-specific differences in counts. We also computed age-adjusted incidence rates of invasive breast cancer in order to examine and quantify the impact of differences between the population data sources.

**Results:**

Differences between year 2000 DOF projections and year 2000 census counts varied by county and age and ranged from underestimates of 60% to overestimates of 64%. For Marin County, the DOF underestimated the number of nHW women aged 45 to 64 years by 32% compared to the 2000 US census. This difference produced a significant 22% discrepancy between breast cancer incidence rates calculated using the two population data sources. In Los Angeles and Santa Clara counties, DOF-based incidence rates were significantly lower than rates based on census data. Rates did not differ significantly by population data source in the remaining seven counties examined.

**Conclusion:**

Although year 2000 population estimates from the DOF did not differ markedly from census counts at the state or county levels, greater discrepancies were observed for race-stratified, age-specific groups within counties. Because breast cancer incidence rates must be calculated with age-specific data, differences between population data sources at the age-race level may lead to mis-estimation of breast cancer incidence rates in county populations affected by these differences, as was observed in Marin County. Although intercensal rates based on population projections are important for timely breast cancer surveillance, these rates are prone to bias due to the error of closure between population projections and decennial census population counts. Intercensal rates should be interpreted with this potential bias in mind.

## Introduction

**Figure 1:**
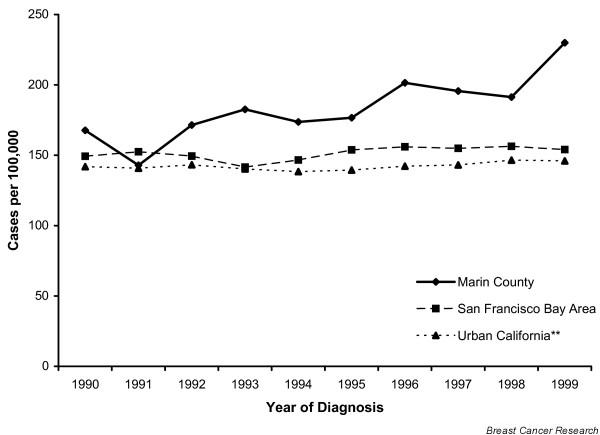
Breast cancer incidence in Marin County, California, and surrounding areas based on Department of Finance population projections for 1990 to 1999. Rates are age-adjusted to the 2000 US standard population, based on population data unadjusted to the 2000 US census. **Case data from the California Cancer Registry.

From the inception of the Surveillance, Epidemiology, and End Results (SEER) national cancer registry network in 1973, Marin County, California, a small county near San Francisco, has consistently reported higher than average annual incidence rates of breast cancer. Averaged from 1973 to 1999, Marin County reported the highest overall breast cancer incidence rate of the 199 counties included in the SEER database (based on the SEER 9 November 2001 submission released April 2004) [1]. In recent years, reports of rapidly increasing breast cancer rates in Marin County attracted public and media attention. These reports suggested that overall age-adjusted incidence rates of invasive breast cancer in non-Hispanic white (nHW) women living in Marin County had increased approximately 60% between 1990 and 1999, as compared to 5% in surrounding regions (Fig. 1) [2]. These trends have resulted in Marin County having one of the highest incidence rates reported in the world and have prompted public and scientific concern.

Several possible explanations have been suggested for these breast cancer incidence patterns. Overall, the socio-demographic profile of most women living in Marin County would suggest a higher prevalence of women with known risk factors for breast cancer: relatively high proportions of the county population are of nHW ethnicity and college-educated, and the county has a median household income almost double the national average (derived from data from Population estimates, 2000 Census of Population and Housing, 1990 Census of Population and Housing, Small Area Income and Poverty Estimates, County Business Patterns, 1997 Economic Census, Minority- and Women-Owned Business, Building Permits, Consolidated Federal Funds Report, 1997 Census of Governments [3]). In addition, women living in Marin County have fewer children, report a later age at first childbirth, and have higher rates of alcohol consumption than most areas of California, all of which correspond to an increased risk of developing breast cancer [4-6].

Another possible explanation for the observed increase in breast cancer incidence in Marin County during the 1990s involves error in population estimates used in the calculation of cancer rates. Intercensal population estimates, as are used to calculate breast cancer incidence rates for Marin County, are used by a variety of health surveillance organizations nationwide. In order to track changes in the occurrence of health outcomes in a timely manner, disease registries, vital statistics agencies, and local health departments must rely on timely estimates of annual population size; however, for most locales in the United States, the population is counted only once every 10 years as part of the national census. Population estimates for intercensal years are projected from census counts from the most recent decennial census along with other governmental data (e.g., vital statistics and immigration records), and are subject to adjustment after the release of data from the subsequent census. The discrepancy between year 2000 population data from the 2000 census and population projections for the year 2000 based on the 1990 census is known as the 'error of closure'.

In California, intercensal population projections are available from two sources, the US Census Bureau and the California Department of Finance (DOF). Although it is uncertain which agency produces more accurate population projections, most California health agencies rely on data from the DOF, perhaps because the methodology used by the census includes little county-specific information, because significant flaws in Census Bureau-produced estimates have been cited in the past, and because the DOF incorporates additional county- and state-specific information into population projections [7-9]. In order to project the size of the population of California by county, gender, race/ethnicity, and 1 year age increments, for the next 50 years, the DOF not only uses data from the most recent national census, but also enhanced state data resources, such as state records of drivers license change of address transactions, migration patterns based on previous censuses, ethnic group-specific fertility rates, information from the Department of Corrections regarding the capacity and flow of prisoners through facilities, and information from the Pentagon to predict military base closures and reassignments. The DOF also makes adjustments to all intercensal population projections dating back to the previous national census with the release of new national census counts [9,10].

Despite the detailed algorithm used by the DOF to project the distribution and size of the California population, these projections are subject to the same limitations as intercensal population figures generated by the Census Bureau; the risk of inaccuracy increases as annual estimates become more temporally removed from the most recent census. Furthermore, estimates for small areas, or certain age, gender, and racial/ethnic groups are prone to even larger biases due to algorithm inaccuracies: areas with a high growth rate, a large population of retirees, or a large population of foreign-born individuals are likely to be underestimated, while areas with high poverty, and areas with a negative growth rate are likely to be overestimated [7].

The following analysis was conducted to assess the impact on breast cancer incidence rates of the error of closure in stratified DOF projections, 10 years removed from the most recent national census. The goals of this analysis were: to examine how closely population estimates for the year 2000 from the California DOF correlated overall and for selected population strata with counts from the 2000 US census; and to assess how breast cancer incidence rates in selected California counties could be affected by the error of closure between DOF estimates and census counts.

## Materials and methods

### Data sources and study population

At the time of this analysis, 10 counties in California participated in the National Cancer Institute's SEER program (Alameda, Contra Costa, Marin, San Francisco, San Mateo, Monterey, San Benito, Santa Clara, Santa Cruz, and Los Angeles). Data on incident invasive breast cancers diagnosed between 1999 and 2001 in these counties were accessed with public-use SEER data files (based on the SEER 11 Sub for Expanded Races November 2003 submission released April 2004) [1].

Age, sex, and race/ethnicity-specific population data for the year 2000 for counties under analysis were obtained from the DOF and the US Census Bureau [9,11]. Year 2000 DOF estimates used in these analyses were projected based on the 1990 census and were not adjusted to the 2000 census, although DOF data adjusted to the 2000 census are now available. Year 2000 data from the US Census represent actual year 2000 counts.

Analyses were limited to nHW women. We limited the population to this group to avoid confounding by race/ethnicity; because breast cancer incidence rates are higher among nHW women than among women of any other race, to include breast cancer incidence estimates for Los Angeles County (where only 31% of the population is nHW) and estimates for Marin County (where 79% of the population is nHW) in the same analysis, without accounting for race, could be misleading [5]. There are, however, important differences in the way the DOF and the census categorize race/ethnicity. The 2000 US Census allowed individuals to report up to six distinct ethnicities concurrently to categorize themselves, whereas the DOF stratified the population into five mutually exclusive race categories (white, Hispanic, African-American, Asian Pacific Islander, and American Indian) [10,11]. To control for these differences in race categorization, a US census dataset with bridged race categories was used [12].

### Comparison of population data

Year 2000 population data from the DOF and the census were compared overall and stratified by county, gender, and age group. We examined these stratified groups in order to identify and describe those most likely to be impacted by discrepancies in population estimates.

For all comparisons, 2000 census data were chosen as the standard. Percent differences between data sources can thus be interpreted as the percent by which DOF estimates overestimate or underestimate corresponding census counts. Very small percent differences are to be expected due to the fact that census estimates are based on the population as of 1 April 2000, whereas DOF estimates are based on the population as of the middle of the year [10,11].

### Analysis of incidence rates

For the comparison of breast cancer incidence rates, we included cases of invasive breast cancer (classified as 50.0–50.9 by the International Classification of Diseases; Oncology, 2^nd ^edition) diagnosed between the years 1999 and 2001 [13]. County-specific incidence rates were age-adjusted using direct-standardization methods, adjusting to the 2000 US standard population [14]. Incidence rates based on year 2000 DOF population estimates, and their corresponding 95% confidence intervals, were compared to incidence rates based on year 2000 census counts for each county under analysis.

Standardized rate ratios were calculated to compare DOF-based and census-based incidence rates for all counties under analysis. Census-based rates were used as the reference in all regression models, such that rate ratios derived from each of the 10 county-specific models describe the influence of discrepancies between rate denominators independent of the rate numerator.

## Results

Overall, the DOF estimated the size of the year 2000 California population to be 2.3% larger than was counted by the census. When restricted to nHW women in the 10 counties under analysis, DOF population estimates exceeded census counts by approximately 2.9%, ranging from 5.7% below census counts in San Francisco County to 10.4% above census counts in San Benito County. Table 1 summarizes discrepancies between population data sources by age strata and county.

**Figure 2:**
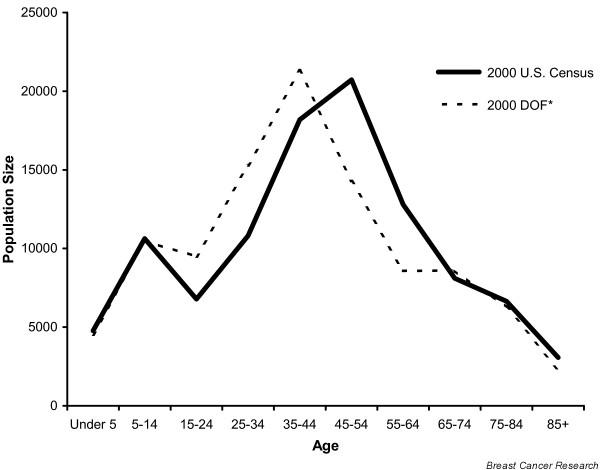
Marin County, California, population estimates for non-Hispanic white women by age and population data source (2000). DOF, Department of Finance.

When further stratified by age group, DOF and census county population data for nHW women differed more significantly, although patterns of overestimation and underestimation across age strata differed by county. Percent differences between the two population data sources ranged from <0.1% to 64.1%. Discrepancies by age group were largest in San Francisco County, where the percent difference between DOF and census data was more than 30% in 4 of 10 age groups for nHW women (ranging from -60.3% to 64.1%), and in Marin County, where percent differences also exceeded 30% in 4 of 10 age groups (ranging from -32.9% to 41.4%). More importantly, however, were discrepancies between population estimates for age groups with the highest incidence of breast cancer; among nHW women aged 45 years and older, the most substantial population data discrepancies were in Marin County, where DOF population projections for nHW women aged 45 to 64 years fell below census estimates by approximately 31.7% and in San Benito County, where DOF estimates for the 55 years and older population exceeded census estimates by 30.6%. Age-specific discrepancies for Marin County are plotted in Fig. 2.

Direct comparison of year 2000 DOF- and census-based age-adjusted incidence rates by county revealed significant differences in estimated incidence rates by population source in three of the ten counties under analysis (Table 2). Breast cancer incidence rates in Santa Clara and Los Angeles counties were significantly lower when based on DOF county population estimates compared to census county population data, adjusting for age: the DOF-based rate was 143.4, 95% CI = (137.5–149.5) versus the census-based rate of 158.6, 95% CI = (152.0–165.4) in Santa Clara; and the DOF-based rate was 153.8, 95% CI = (150.7–156.9) versus the census-based rate of 161.0, 95% CI = (157.8–164.3) in Los Angeles. Marin County was the only county where the DOF-based rate was significantly higher than the census-based rate: the DOF-based rate was 213.6, 95% CI = (198.4–229.9) versus the census-based rate of 175.8, 95% CI = (163.2–189.5)). The DOF-based rates for Marin County were approximately 22% higher than census-based rates based on the same numerators.

## Discussion

These analyses have explored the extent to which use of intercensal population projections, extrapolated and 10 years removed from the 1990 census, may have biased breast cancer incidence rates reported in California in the 1990s. DOF-based incidence rates for Marin, Santa Clara, and Los Angeles counties were found to differ significantly from census-based incidence rates: county-specific DOF-based rates were lower than census-based rates in Santa Clara and Los Angeles counties, but higher than census-based rates in Marin County.

Direct comparison of year 2000 DOF and census population data revealed accuracy of DOF projections at the state level, although joint stratification of population estimates by county, gender, race/ethnicity, and age introduced greater discrepancy between population data sources. These discrepancies between stratified population estimates were significant enough to lead to notable differences in breast cancer incidence rates, and can be expected to have a notable effect on other statistics based on DOF intercensal estimates not adjusted to the 2000 census. For example, in the case of San Francisco County, substantial overestimation of the 5–14 year old nHW female population (64.1%) by the DOF compared to the 2000 US census, while having a negligible effect on county breast cancer incidence rates due to the negligible rate of breast cancer among this age group, may be anticipated to have a notable impact on childhood cancer rates.

The fact that differences between population data sources had a significant effect on breast cancer incidence rates in the two largest counties analyzed (Los Angeles, population 9,519,338, and Santa Clara, population 1,682,585) and the second smallest county analyzed (Marin, population 247,289) suggests that population size is not responsible for variation between census and DOF data. Indeed, no pattern of deviation between the two population data sources is discernable by county size, age distribution, or county urban/rural status. It is possible that methods used by the census and methods employed by the DOF are differentially effective among different populations, or that differing levels of domestic migration explains the discrepancies between these population data sources. The source of these discrepancies, however, remains unknown and was beyond the scope of this analysis.

**Figure 3:**
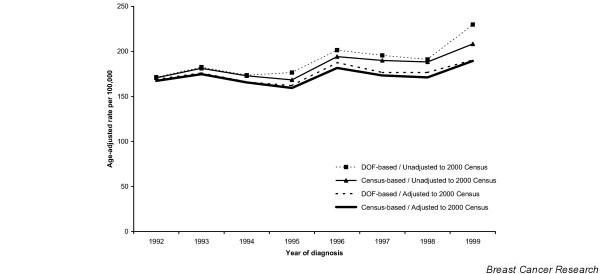
Breast cancer incidence in Marin County, California, by population data source, and adjustment 2000 US census, for 1992–1999.

One limitation to the applicability of this analysis is that DOF intercensal population projections are not used as widely as projections provided by the US census, as DOF projections are only available for counties in the state of California; however, problems similar to those noted in this analysis have been noted with the application of census projections [7]. Errors in intercensal population projections provided by the US census for the years 1991 to 1999 were recently implicated as a source of significant overestimation of racial disparities in cancer incidence rates [7,15]. In Marin County, US census intercensal population projections were subject to error of closure problems similar to those identified in DOF projections; census projections, unadjusted to the 2000 US census, substantially underestimated the high risk group of Marin County nHW women aged 45 to 74 years (data not shown), resulting in an overestimation of the overall incidence rate of breast cancer in the latter years of the 1990s (Fig. 3). Thus, it is likely that similar conclusions would have been reached had census intercensal projections been used rather than DOF intercensal projections. Although methodology used to generate intercensal population projections by both the DOF and the Census Bureau is intricate, complex, and complete, both agencies have produced inaccurate projections. These error of closure problems mean that population data, and incidence rates based on these data, become less reliable as they become further removed from the most recent census.

## Conclusion

The California Cancer Registry, as well as county and local governmental agencies and a broad range of community organizations, must rely on intercensal population projections to estimate health trends, allocate resources, and establish priorities with respect to the populations they serve. Timely surveillance requires that intercensal population projections be used to generate population-based rates and trends as soon as reliable incidence counts become available. This analysis, however, demonstrates that intercensal population projections can differ substantially from later decennial census counts. Although it is unrealistic to recommend that disease surveillance be paused in intercensal years, these data remind us that population denominator quality can have a major impact on the interpretation of health statistics. Health agencies must judge whether aberrant health trends should be acted upon prior to the release of population information that could inform the accuracy of population projections, a process that could take five years or more. The gravity of this problem is magnified in the case of diseases like breast cancer that are the focus of public concern and activism, which intensifies demand for information and public health action.

The results of this analysis support the need for a restructuring of population estimation procedures; perhaps more frequent collection of population counts, particularly in regions experiencing high levels of migration. A 10-year period between population censuses is problematic for accurate projection of the age/gender/race-specific yearly population counts needed for health tracking. Alternatively, government agencies producing population projections would benefit from improvements in ways to make more accurate assumptions regarding the growth and distribution of the population. At the least, more health agencies should develop better ways to describe and quantify the uncertainties in population projections and related bias to consumers of health statistics.

## Abbreviations

DOF = Department of Finance; nHW = non-Hispanic White; SEER = Surveillance, Epidemiology, and End Results;

## Competing interests

The author(s) declare that they have no competing interests.

## Authors' contributions

AIP completed all analyses and led the writing. R.R. Ereman was the supervising researcher on this project, helped conceive of the study idea and supervised the writing process. C.A. Clarke assisted with the conception of the study analysis and advised the writing. All authors helped review drafts of the manuscript and interpret study findings.

## Figures and Tables

**Table 1:** Percent difference between DOF and census population estimates for non-Hispanic white women by age (2000)^a^

Age (years)	Alameda	Contra Costa	Marin	San Francisco	San Mateo	Monterey	San Benito	Santa Clara	Santa Cruz	Los Angeles
All ages	3.4	3.8	-1.4	-5.7	3.8	4.0	10.4	8.7	4.7	2.1
Under 5	8.5	-1.1	-5.1	23.2	1.5	19.2	6.0	5.2	11.5	0.8
5–14	13.2	<0.1	-1.5	64.1	9.2	27.7	-6.7	10.4	10.8	4.1
15–24	-3.7	26.8	40.0	-54.9	16.2	5.3	41.7	6.0	-3.8	-12.8
25–34	-19.5	-0.9	41.4	-60.3	-21.9	-11.4	15.4	-4.4	1.7	-11.1
35–44	9.2	-4.4	17.3	40.7	2.3	14.4	-8.0	14.8	4.3	13.6
45–54	10.9	4.7	-31.1	27.0	8.0	-2.5	-0.6	12.7	5.5	9.1
55–64	13.9	13.1	-32.9	11.4	14.5	-3.8	24.0	20.1	7.6	9.1
65–74	3.9	9.7	5.9	7.7	10.6	-2.3	47.0	13.0	8.8	-0.3
75–84	-4.4	-4.8	-5.8	9.1	2.6	-3.8	24.6	-2.1	5.2	-3.0
85+	-4.9	-10.3	-24.7	20.8	-2.8	-1.5	25.0	-14.5	3.3	2.8

^a^Percent difference = ([DOF - census]/census) × 100. DOF, Department of Finance.

**Table 2:** DOF-based versus census-based breast cancer incidence rates among non-Hispanic white women (1999–2001)

County	Average cases per year	DOF-based incidence rate (95% CI)^a^	Census-based incidence rate (95% CI)^a^	Standardized rate ratio
Alameda	575	143.3 (136.6–150.4)	151.8 (144.6–159.2)	0.94
Contra Costa	592	158.0 (150.7–165.7)	164.9 (157.2–172.9)	0.96
Marin	245	213.6 (198.4–229.9)	175.8 (163.2–189.5)	1.22^b^
San Francisco	296	140.3 (131.0–150.7)	159.8 (149.2–171.0)	0.88
San Mateo	406	152.3 (143.7–161.4)	163.4 (154.2–173.2)	0.93
Monterey	172	152.7 (139.7–166.9)	150.7 (137.7–164.8)	1.01
San Benito	19	114.0 (85.9–149.0)	138.7 (104.6–182.0)	0.82
Santa Clara	743	143.4 (137.5–149.5)	158.6 (152.0–165.4)	0.90
Santa Cruz	161	148.2 (135.1–162.4)	157.8 (143.8–173.0)	0.94^b^
Los Angeles	3587	153.8 (150.7–156.9)	161.0 (157.8–164.3)	0.96^b^

^a^Incidence rates are expressed per 100,000 women based on year 2000 population denominators. Data are adjusted to the 2000 US standard. ^b^p < 0.05. DOF, Department of Finance.
